# Search strategies to identify reports on “off-label” drug use in EMBASE

**DOI:** 10.1186/1471-2288-12-190

**Published:** 2012-12-29

**Authors:** Bita Mesgarpour, Markus Müller, Harald Herkner

**Affiliations:** 1 Department of Clinical Pharmacology, Medical University Vienna, General Hospital, Währinger Gürtel 18-20, Vienna, 1090, Austria; 2 Department of Emergency Medicine, Medical University Vienna, General Hospital, Währinger Gürtel 18-20, Vienna, 1090, Austria

**Keywords:** Off-label use, Information retrieval, EMBASE, MEDLINE, Sensitivity

## Abstract

**Background:**

Medications are frequently prescribed outside their regulatory approval (off-label) by physicians particularly where appropriate therapies are not available. However, the risk/benefit ratio of drugs in off-label use needs to be critically appraised because it may differ from approved on-label usage. Therefore, an extensive exploration of current evidence on clinical data is well-advised. The objective of this study was to develop a search strategy that facilitates detection of the off-label drug use documents in EMBASE via OvidSP.

**Methods:**

We constructed two sets of gold standards from relevant records to off-label drug use by a sensitive search of MEDLINE and EMBASE. Search queries, including search words and strings, were conceived based on definition of off-label use of medications as well as text analysis of 500 randomly selected relevant documents. The selected terms were searched in EMBASE (from 1988 to 2011) and their retrieval performance was compared with the gold standards. We developed a sensitivity-maximizing, and a sensitivity- and precision-maximizing search strategy.

**Results:**

From 4067 records relevant to off-label drug use in our full gold standard set, 3846 records were retrievable from EMBASE. “off label*.af.” was the most sensitive single term (overall sensitivity 77.5%, sensitivity within EMBASE 81.9%, precision 88.1%). The highest sensitive search strategy was achieved by combining 36 search queries with overall sensitivity of 94.0% and precision of 69.5%. An optimal sensitive and precise search strategy was yielded precision 87.4% at the expense of decreasing overall sensitivity to 89.4%.

**Conclusion:**

We developed highly sensitive search strategies to enhance the retrieval of studies on off-label drug use in OvidSP EMBASE.

## Background

Pharmacotherapy is usually based on drugs that are approved for specific indications, dosages, routes of administration, or populations. However, administration of drugs outside these approved purposes is possible and denoted “off-label drug use”. Off-label drug use is common practice, but generally suffers from a lack of sufficient evidence on risk/benefit assessment [1-4]. For some drugs off-label use is clinically more important than for approved purposes. For example, in a recent report off-label use of factor VII was found to be more frequent than its on-label indications [5]. Nonetheless, there is increased effort to provide an evidence base for off-label use of drugs. Therefore, it appears important to explore the best strategy to find as many relevant studies as possible.

Free access to MEDLINE through the PubMed interface and its broad coverage makes it the first-choice database in biomedical literature [6,7]. Excerpta Medica Database (EMBASE) is also considered as a major bibliographic database in biomedicine but it is only available by subscription [8,9]. EMBASE covers pharmacology, pharmaceutical science and clinical research as its main areas of interest. In comparison with MEDLINE, it provides more extensive coverage of European and non-English language publications [10] as well as conference abstracts [9].

Accordingly, systematic searches restricted to MEDLINE only are generally not advised, because of potential introduction of retrieval bias [11-14]. Moreover, each bibliographic database has different indexing practice and thesaurus system. For example, records in MEDLINE are indexed using the National Library of Medicine’s controlled vocabulary Medical Subject Headings (MeSH®) and EMBASE uses a thesaurus called EMTREE, which includes medical terms, drug names, acronyms, MeSH® headings and spelling variations. Therefore, even within one interface like OvidSP a search strategy is specific for a particular database. It is unclear whether a search strategy can be directly translated for applying in other databases, without loss of sensitivity or precision [15]. This may result in missing studies or retrieving many irrelevant documents. In a recent study, we found that MEDLINE did not cover 46% of off label drug use studies [16]. As a consequence we set out here to report the retrieval properties of selected search queries and combined queries for identifying off-label drug use studies in EMBASE.

## Methods

Our methods are detailed elsewhere [16]. Briefly, we did a systematic and sensitive search in MEDLINE and EMBASE through OvidSP (from 1948 and 1988, respectively; last updated in 28 February 2011) to find studies on off-label use of drugs. We constructed two sets of gold standards: the external or full set gold standard contained the whole relevant records retrieved from these two databases; the internal gold standard was the subset of documents indexed in EMBASE. Search queries and strategies were then created and tested for their ability to retrieve relevant records in EMBASE. We assigned a search query as one line in a search strategy. It is usually a text string containing the exact sequence of words and/or characters, and may include Boolean or proximity operators. Consequently, a search strategy consisted of several search queries connected with Boolean operators.

To construct a comprehensive set of possible search queries, we expanded our list of controlled vocabulary search terms or subject headings (EMTREE in EMBASE), text words and strings by text analysis of 500 random documents in the gold standard full set. We applied frequently used fields in OvidSP EMBASE for searching like “.af.” for all searchable fields, “.ab.” for abstract, “.ti.” for title and “.mp.”, which restricts OvidSP's search to the text of title, abstract, subject headings, heading word, drug trade name, original title, device manufacturer or drug manufacturer name. We also used the Boolean operators “OR”, “NOT”, and proximity operator “ADJ[Number]”, as well as truncation and wildcards.

We considered documents as relevant to off-label drug use, if they referred to any human drug outside the approval purposes, in terms of different dose, indication, route of application or for another age group. We did not apply any language restrictions, but method-wise we excluded book series, videos, errata, and corrections. We created a Microsoft (MS) Access database to store and manage all records retrieved by the search queries.

We compared the retrieval performance of each candidate term and combination of queries with the internal gold standard reference set by determining their sensitivity, precision and number needed to read (NNR). We chose an external gold standard in addition to the EMBASE gold standard to establish the general performance of our search strategies, supplementary to the performance which is driven by EMBASE indexing. Hence, we defined “overall sensitivity” as the number of relevant records in EMBASE retrieved by a search query/strategy divided by the total number of relevant records in the full gold standard set.

Sensitivity for a given search is defined as the proportion of relevant records retrieved from the database:

$$\mathrm{Sensitivity}=\frac{\mathrm{number}\;\mathrm{of}\;\mathrm{relevant}\;\mathrm{records}\;\mathrm{in}\;\text{a}\;\mathrm{database}\;\mathrm{retrieved}\;\mathrm{by}\;\text{a}\;\mathrm{search}\;\mathrm{strategy}}{\mathrm{total}\;\mathrm{number}\;\mathrm{of}\;\mathrm{relevant}\;\mathrm{records}\;\mathrm{in}\;\mathrm{the}\;\mathrm{database}}\times \;100$$

Precision is the proportion of relevant records retrieved in the search, which is equivalent to positive predictive value:

$$\mathrm{Precision}=\frac{\mathrm{number}\;\mathrm{of}\;\mathrm{relevant}\;\mathrm{records}\;\mathrm{retrieved}\;\mathrm{by}\;\text{a search}\;\mathrm{strategy}}{\mathrm{total}\;\mathrm{number}\;\mathrm{of}\;\mathrm{records}\;\mathrm{retrieved}\;\mathrm{by}\;\mathrm{the}\;\mathrm{search}\;\mathrm{strategy}}\times \;100$$

The NNR refers to the number of non-relevant records that one has to screen to find one of relevance [16]:

$$\mathrm{NNR}=\frac{1}{\mathrm{precision}}\times \;100$$

We ultimately aimed at developing a highly sensitive search strategy (HSSS) with two optimized versions: (1) a sensitivity-maximizing version and (2) a sensitivity- and precision-maximizing version.

For the development of search strategies to optimize sensitivity or precision, we tested all combination of search queries by using an algorithm programmed in C++. The search strategy with the highest balance of sensitivity and precision was developed by creating the scatter plot of precision versus sensitivity and calculating their best trade-off for the combined queries.

For hypothesis testing we used the Fisher's exact test for independent data and the McNemar test for correlated data with a two-sided p-value <0.05 to declare statistical significance.

## Results

### Retrieved documents

**Figure 1:**
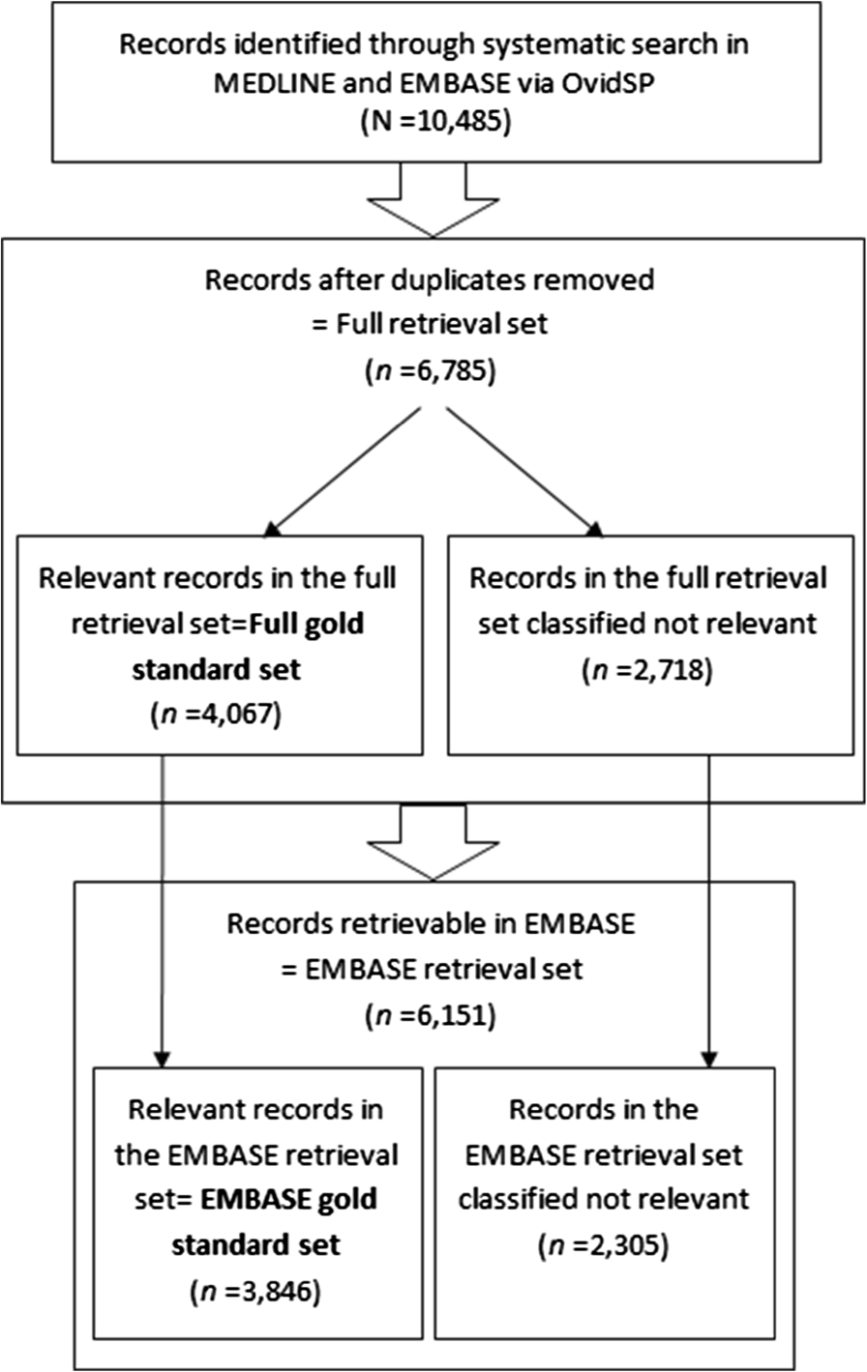
Flow diagram of retrieval and screening process for the gold standard set.

From 6,785 unique records, which were retrieved by a systematic search in MEDLINE and EMBASE via OvidSP, we classified 4,067 as relevant records to off-label drug use. This constituted our full gold standard set (Figure 1). This set included 3,846 records retrievable within EMBASE, which was 62.5% of total retrieved records in EMBASE; among them, 3,480 (90.5%) were published between years 2001-2011.

### Search results

We evaluated the performance of 77 search queries and their combinations. In Table 1, we present the performance of the 15 search queries with the highest sensitivity (see Additional file 1 for the full list). “off label*.af.” yielded the highest sensitivity among the single terms (overall sensitivity 77.5%, sensitivity within EMBASE 81.9%, precision 88.1%). Most of the top performing search queries had NNR of 1.1, which indicates that almost every abstract read classifies on off-label drug use. Truncation “off label” resulted in retrieving 35 more records, of which 31 (88.6%) were relevant. It retrieved more relevant records than “(off adj2 label*).mp.”, although the latter retrieved two unique records.

**Table 1:** **Sensitivity, precision and number needed to read (NNR) of the 15 search queries in OvidSP EMBASE with the highest sensitivity** (see the complete list in Additional file 1)

**Queries**	**Number of relevant records retrieved**	**Number of records retrieved**	**Sensitivity/full set (%)**	**Sensitivity/EMBASE set (%)**	**Precision (%)**	**NNR**
off label*.af.	3150	3577	77.5	81.9	88.1	1.1
(off adj2 label*).mp.	3131	3589	77.0	81.4	87.2	1.1
(off adj1 label*).mp.	3130	3567	77.0	81.4	87.7	1.1
off label*.mp.	3128	3555	76.9	81.3	88.0	1.1
off label.af.	3119	3542	76.7	81.1	88.1	1.1
(off adj1 label).mp.	3119	3544	76.7	81.1	88.0	1.1
(off adj2 label).mp.	3119	3547	76.7	81.1	87.9	1.1
off label.mp.	3117	3540	76.6	81.0	88.1	1.1
"off label*".ab,ti.	2306	2696	56.7	60.0	85.5	1.2
off label.ab,ti.	2293	2679	56.4	59.6	85.6	1.2
"off label*".ab.	1882	2224	46.3	48.9	84.6	1.2
off label.ab.	1870	2208	46.0	48.6	84.7	1.2
(drug adj2 label adj2 us*).af.	1587	1719	39.0	41.3	92.3	1.1
(drug adj1 label adj1 us*).af.	1581	1710	38.9	41.1	92.5	1.1
"off label drug us*".af.	1580	1677	38.8	41.1	94.2	1.1

^**¶**^ADJn is a positional operator to retrieve records that contain the terms (in any order) within a specified number (n) of words of each other. The adj1 operator finds two terms next to each other in any order. The adj2 operator finds terms in any order and with one word (or none) between them.

The overall sensitivity of search query "off label" and its truncation increased by 10.4% when we broadened their field from abstract (.ab.) to abstract and title (.ab,ti.). The most specific subject heading was "off label drug use". Exploding this to all its subheadings, "off label drug use".sh., resulted in 36.8% overall sensitivity (sensitivity within EMBASE 38.9%). Truncating the text string "off label drug use" and then applying all field (.af.) slightly increased its overall sensitivity to 38.1% and 38.8%, respectively.

We retrieved substantial non-relevant records after searching “label adj1 us*.af.” consisted of the following terms: “using label-free” indicating different types of label-free proteomics technologies; “extra-label use” referring the off-label use of veterinary drugs; “open-label use” as a type of study; “spin label using” specifying a tool to study the structure of proteins and biological membranes; and “food/nutritional label use” ascribing the information provided on food products.

To improve the precision of search term “inappropriate us*”, we excluded the records about rational drug use and polypharmacy by NOTing out “antibiotic* or antimicrobial”. The precision of search term “unlicense*.af.” was improved by 30% and its NNR declined about two fold by eliminating retrieval of irrelevant content. It resulted by NOTing out an ORed string of 31 search terms prevalent in its irrelevant records retrieved (see Additional file 1).

To find the relevancy of some retrieved records, particularly those with no abstract such as the letters we had to read their full text. Whenever we detected some relevant editorial, letter or commentaries on other studies, we checked the retrieval status of cited study, consequently. In some cases, we found that we had not retrieved the relevant cited study through our search queries. For example, we retrieved an editorial record [17] on a study of Acharya and associates [18] about off-label use of ranibizumab in uveitis. However, none of our search queries could retrieve the respective original article, despite our text analysis showed that the term “off-label” has been used four times in the full-text of Acharya paper [18].

### Final search strategies

We constructed the best performing search strategy in terms of maximized sensitivity by “OR” combination of 32 search queries. This resulted in an overall sensitivity of 93.98% (99.38% within EMBASE) and a precision of 69.48% (Table 2). Enhancement of sensitivity and precision after incrementally ORing new search queries is shown as cumulative sensitivity and precision in Table 2.

**Table 2:** Highly Sensitive Search Strategy for identifying off-label drug use reports in OvidSP EMBASE: sensitivity-maximizing version

	**Search query**	**Cumulative number of relevant records retrieved**	**Cumulative number of records retrieved**	**Cumulative sensitivity/full set (%)**	**Cumulative sensitivity/EMBASE set (%)**	**Cumulative Precision (%)**
1	off label*.af.	3150	3577	77.45	81.90	88.06
2	(off adj1 label).mp.	3152	3581	77.50	81.96	88.02
3	(drug adj2 label adj2 us*).af.	3153	3617	77.53	81.98	87.17
4	unlicense*.af.	3279	4320	80.62	85.26	75.90
5	unapprove*.af.	3445	4635	84.71	89.57	74.33
6	(label adj3 indication*).af.	3450	4667	84.83	89.70	73.92
7	off li?en?e*.af.	3509	4742	86.28	91.24	74.00
8	((no* licen?ed for adj3 use*) not now licen?ed).af.	3560	4825	87.53	92.56	73.78
9	((inappropriate us* and indication) not (antibiotic* or antimicrobial)).af.	3609	4964	88.74	93.84	72.70
10	((appropriate* adj3 prescri*) and indication).af.	3648	5081	89.70	94.85	71.80
11	(outside adj3 licen?e*).af.	3665	5110	90.12	95.29	71.72
12	unlabel* us*.af.	3692	5140	90.78	96.00	71.83
13	labeled indication*.af.	3703	5169	91.05	96.28	71.64
14	(inappropriate indication*).af.	3719	5294	91.44	96.70	70.25
15	nonapprove*.af.	3737	5337	91.89	97.17	70.02
16	registered indication*.af.	3752	5367	92.25	97.56	69.91
17	offlabel*.af.	3757	5372	92.38	97.69	69.94
18	(out* adj4 licen?ed indication*).af.	3759	5376	92.43	97.74	69.92
19	(unlabel* adj3 indication*).af.	3767	5384	92.62	97.95	69.97
20	non fda approve*.af.	3780	5411	92.94	98.28	69.86
21	((no* licen?ed for adj3 indication*) not now licen?ed).af.	3793	5425	93.26	98.62	69.92
22	(appropriate indication adj3 us*).af.	3796	5432	93.34	98.70	69.88
23	(be???d* adj2 licen?ed indication*).af.	3799	5436	93.41	98.78	69.89
24	(us* without adj2 indication*).af.	3804	5445	93.53	98.91	69.86
25	(prescri* outside adj4 guideline*).af.	3808	5450	93.63	99.01	69.87
26	(out of label).af.	3810	5466	93.68	99.06	69.70
27	(improper adj1 indication*).af.	3812	5472	93.73	99.12	69.66
28	(inappropriate adj5 indication adj2 us*).af.	3814	5475	93.78	99.17	69.66
29	no* appropriate indication*.af.	3815	5482	93.80	99.19	69.59
30	(non evidence base* us*).af.	3818	5487	93.88	99.27	69.58
31	without proper indication*.af.	3821	5498	93.95	99.35	69.50
32	(or/1-31) or (drug* without adj2 indication*).af.	3822	5501	93.98	99.38	69.48

Combinations of search queries with the best sensitivity are ranked in descending order of number of relevant records.

Overall sensitivity and precision of the EMBASE HSSS (sensitivity maximized) was higher in the recent years (2001-2011) compared to years 1988-2000 (95.6% vs 79.3% and 75.0% vs 40.8%, p<0.001 for both), while sensitivity within EMBASE remained approximately constant (99.5% vs 99.4%; p=0.99, Fisher exact test).

We also tested the EMBASE HSSS (sensitivity maximized) in the full gold standard set excluding 500 records that used to develop our strategies and received virtually the same results.

To evaluate the retrieval function of off-label drug use search strategy developed for a different database, we further tested our published search strategy for OvidSP MEDLINE [16] in OvidSP EMBASE. The retrieval implications of re-running our published highly sensitive search strategy A for OvidSP MEDLINE (MEDLINE HSSS) in EMBASE yielded an overall sensitivity of 89% and a sensitivity within EMBASE of 94%, precision of this search strategy was 77.7%. Using the EMBASE HSSS (sensitivity maximized) compared with the published MEDLINE HSSS (sensitivity maximized) in EMBASE increased sensitivity from 94% to 99% (p <0.001, McNemar test). Out of 4,067 studies classified relevant in the full set gold standard, 1,942 studies were retrieved through EMBASE HSSS in EMBASE as well as the MEDLINE HSSS in MEDLINE (both sensitivity maximized). By searching in MEDLINE only, 1,880 relevant records were not retrieved whereas by searching in EMBASE only, 222 were not retrieved (p<0.001, McNemar test).

**Figure 2:**
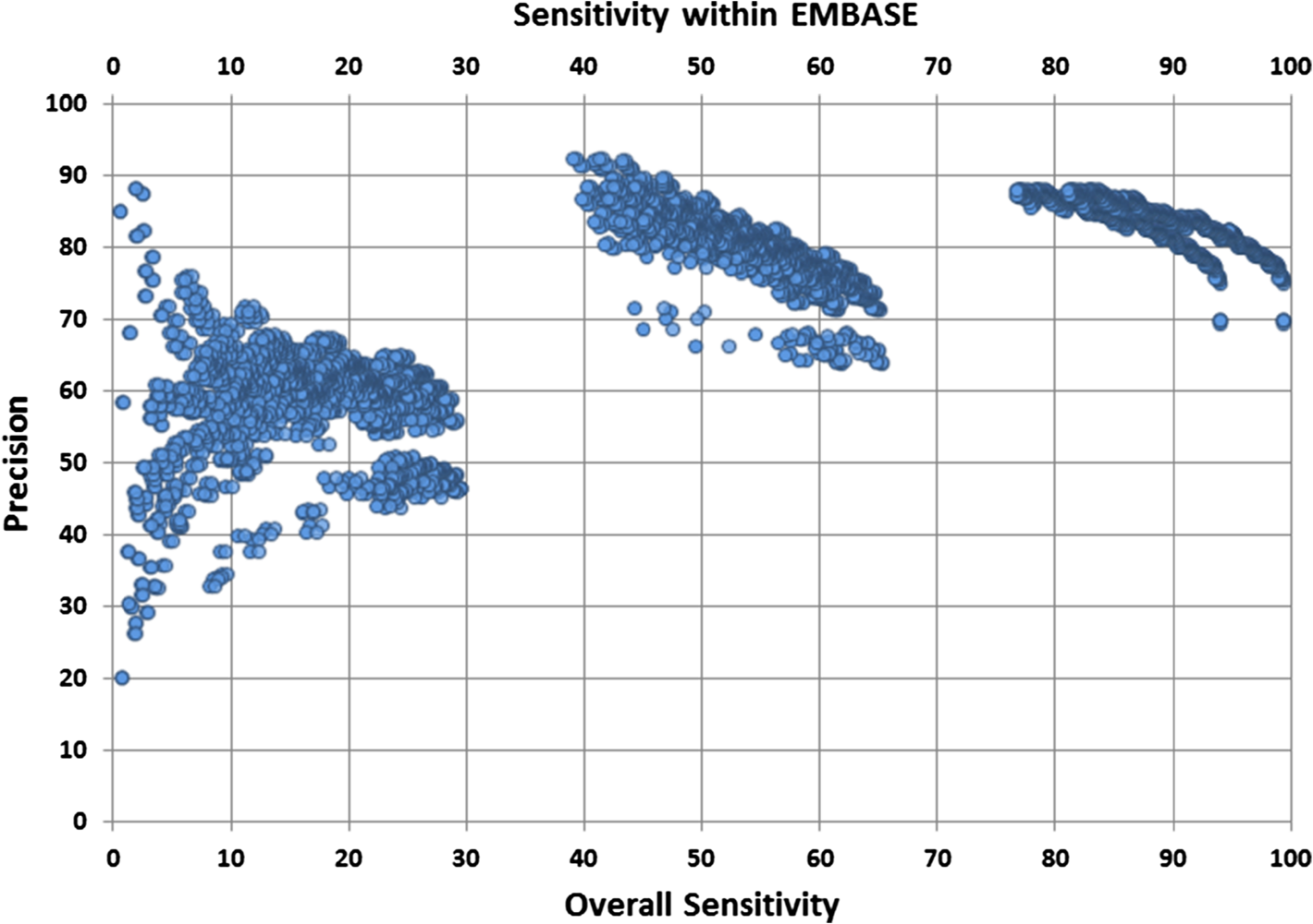
Plot of sensitivities (relevant documents in the overall set (n=4,067) and within EMBASE (n=3,846)) versus precision for different combinations of search queries to detect studies on off-label drug usage in electronic databases.

We plotted precision versus overall sensitivity of search query combinations to optimize sensitivity and precision (Figure 2). Table 3 shows the most parsimonious search strategy amongst 15 strategies where sensitivity was optimized for high precision (see Additional file 2). The combination of 22 search queries and query “(stent* or veterinar*).af.” by using a NOT operator eliminated the irrelevant records out of the most optimized sensitive and precise search strategy leading to 5.32% improvement of precision at the cost of a small decrease in sensitivity (overall and within EMBASE 0.37% and 0.39%, respectively).

**Table 3:** Highly sensitive search strategy for identifying off-label drug use reports in OvidSP EMBASE: sensitivity- and precision-maximizing version

	**Search query**	**Cumulative number of relevant records retrieved**	**Cumulative number of records retrieved**	**Cumulative sensitivity/full set (%)**	**Cumulative sensitivity/EMBASE set (%)**	**Cumulative Precision (%)**
1	off label*.af.	3150	3577	77.45	81.90	88.06
2	(unlicensed not (unlicensed aide* or unlicensed assist* or unlicensed car* or (Unlicensed adj2 heal*)or unlicensed home* or (killer adj2 cell*) or (unlicensed adj2 individual*) or (unlicensed adj2 nurs*) or (unlicensed adj4 practi*) or (unlicensed adj2 physician*) or (unlicensed adj2 operat*) or (unlicensed adj2 person*) or unlicensed profession* or unlicensed rid* or (unlicensed adj3 staff*) or unlicensed therapist* or (unlicensed adj5 vaccine*) or unlicensed vendor* or (unlicensed adj2 work*) or (unlicensed adj2 employe*) or device*or dentist*or driver or driving or herbal or medical graduate* or motor* or pesticide*or premis*or prostitute*or restaurant*or veterinary or worker*)).af.,	3272	3858	80.45	85.08	84.81
3	(unapprove* adj2 us*).af.	3331	3947	81.90	86.61	84.39
4	(unapprove* adj5 indication*).af.	3367	3986	82.79	87.55	84.47
5	off li?en?e.af.	3425	4053	84.21	89.05	84.51
6	((no* licen?ed for adj3 use*) not now licen?ed).af.	3476	4136	85.47	90.38	84.04
7	(unapprove* adj2 drug*).af.	3493	4181	85.89	90.82	83.54
8	(outside adj3 licen?e*).af.	3511	4211	86.33	91.29	83.38
9	unlabel* us*.af.	3538	4241	86.99	91.99	83.42
10	labeled indication*.af.	3549	4270	87.26	92.28	83.11
11	nonapprove*.af.	3569	4317	87.76	92.80	82.67
12	registered indication*.af.	3585	4348	88.15	93.21	82.45
13	offlabel*.af.	3590	4353	88.27	93.34	82.47
14	(unlabel* adj3 indication*).af.	3598	4361	88.47	93.55	82.50
15	non fda approve*.af.	3611	4388	88.79	93.89	82.29
16	((no* licen?ed for adj3 indication*) not now licen?ed).af.	3624	4402	89.11	94.23	82.33
17	(appropriate indication adj3 us*).af.	3630	4413	89.25	94.38	82.26
18	(be???d* adj2 licen?ed indication*).af.	3633	4417	89.33	94.46	82.25
19	(us* without adj2 indication*).af.	3638	4428	89.45	94.59	82.16
20	(prescri* outside adj4 guideline*).af.	3643	4434	89.57	94.72	82.16
21	(inappropriate adj5 indication adj2 us*).af.	3647	4441	89.67	94.83	82.12
22	(non evidence base* us*).af.	3650	4446	89.75	94.90	82.10
23	(or/1-22) not (stent* or veterinar*).af.	3635	4158	89.38	94.51	87.42

Combinations of search queries with the best optimization of sensitivity and precision are ranked in descending order of number of relevant records. See further search strategies in Additional file 2.

### OvidSP EMBASE characteristics

A substantial change in number of retrieved records in EMBASE was found in early August 2010. 206 duplicated and two triplicated records were retrieved in our last search update. They were indexed in two different accession numbers but mostly in the same entry week. For example, a study by Caron [19] had two records with two accession numbers and similar entry week (17872714 and 2007205136; entry week: 200700) and a study by Daskalaki et al. [20] had two records with two accession numbers and two entry weeks: 18595974 (entry week: 200800) and 2009161626 (entry week: 200900) (see Additional file 3). Moreover, we came across to some other errors such as dup/triplicate records because of typographical errors or more than one translation for non-English titles. For example, a study by Konda et al. [21] recorded in two different titles “Colchicine in dermatology” and “Dosages and administration”. The latter title is wrong and this record has also failed to provide author’s name and had a typo error in start page (202 instead of 201). The correct title was indexed with 10 subject headings and the wrong one with 56 subject headings including “off label drug use” (see Additional file 3). We also found that indexing for documents that are published in more than one journal might be different. It is also the case when a document is published as both a conference abstract and an article. For example, a study by De Jong et al. which is published as a conference abstract [22] and an article [23] was indexed with 15 subject headings where publication type was 'abstract' and with 36 subject headings including “off label drug use” where publication type was 'article'. The abstracts recorded for these two publication types were almost the same (see Additional file 3). However, the database issues presented do not appear to explain much of the performance limitations overall.

The sensitivity and precision of some search queries was different after replacing the “af” field by “mp” in our last update, 28 February 2011. Therefore, we chose the best balance of sensitivity and precision (see Additional file 1). However, running our search queries in 30 January 2012 showed that the discrepancies between these two search fields have been removed. Updating issues during the integration of records from MEDLINE into the OvidSP EMBASE database may explain this (e-mail communication with Ovid Training Department).

## Discussion

We developed highly sensitive search strategies for OvidSP EMBASE to enhance the retrieval of studies on off-label drug use. These highly sensitive search strategies outperformed single search terms, EMTREE terms, and queries as well as search strategies designed for another database.

Top-performing queries were almost the same in EMBASE compared to our published search queries developed for MEDLINE [16]. For example, the queries “off label*.af.”, “(off adj2 label*).mp.” and “(off adj1 label*).mp.” were all top performers in both databases. Three queries “(unlabel* adj3 indication*).af.”, “(unapprove* adj5 prescription).af.” and “(outside adj3 licen?e*).af.” were only selected for EMBASE. We also chose “off label.mp.” because of different retrieval for search fields “af” and “mp” in EMBASE. Furthermore, the most specific subject heading was not exactly identical in two databases: "off label drug use" in EMBASE and "Off-Label Use" in MEDLINE.

Optimized strategies, sensitivity-maximizing version and sensitivity- and precision-maximizing version, in EMBASE had greater overall sensitivity (sensitivity in the full gold standard set) and precision than the comparable MEDLINE strategy. However, the sensitivity within their internal gold standard was almost the same. This can be best explained by different coverage of these databases. For example, a study showed that 47% of 386 Syrian Arab Republic reports indexed in MEDLINE or EMBASE were found exclusively in EMBASE, while 32% from MEDLINE alone and 21% from both of them [24]. Moreover, by the integration of records from MEDLINE into EMBASE, the coverage of EMBASE must have increased [25].

The necessity for developing a specific search strategy adapted to the indexing structure, limits, and special features for each database is extensively reported, although there is no study on retrieval failure [15]. Re-running a highly sensitive search strategy which we developed for identifying off-label drug use in OvidSP MEDLINE [16] in OvidSP EMBASE achieved about 5% less sensitivity compared to our newly developed search strategy, but 8.2% higher precision.

Retrieving relevant studies on off-label drug use might be influenced by many mechanisms such as low quality of off-label use reporting and poor indexing in bibliographic databases. Administration of medication for unapproved purposes is not well defined or properly described in some reports. This might happen when authors are inconsiderate or unfamiliar with the concept. A survey of 95 office-based pediatricians in France showed that they did not recognize the off-label status of 686 out of 745 (92%) drug courses they commonly prescribed [26]. Another study in Northern Ireland on experience and attitudes of healthcare professionals on unlicensed/off-label pediatric prescribing revealed that 41% of 563 respondents were not familiar with the term “off-label medicines” prior to participating in the study [27].

Inadequate relevant keywords (as provided by study authors), inconsistent terminology or ambiguous description in the abstract might lead to the poor indexing, because the whole information contained in the full-text record is not detected by searching in bibliographic databases. Thus, low sensitivity of the specific EMTREE term for off-label drug use studies in EMBASE, "off label drug use".sh., confirmed inconsistent indexing. Another indication of poor indexing is the fact that in some cases secondary papers like letters, commentaries or editorials could be retrieved but not the original papers. Glanville, et al. showed that MEDLINE and EMBASE indexing terms for economic evaluations are not efficient either. They indicated that it could be explained by indexer uncertainty and indexing lapses, in addition to poor reporting by authors [28].

As a result of the low quality of reporting off-label drug use and poor indexing, we will not be able to retrieve all relevant records on off-label drug use by search in bibliographic databases alone, even with the highest sensitive search strategies. Thus, we suggest the implementation of our search strategy already for broad sweep research questions. “Off-label drug use in multiple sclerosis” or “off-label use of beta interferon” might serve as an example.

Search queries included in EMBASE HSSS were the same as MEDLINE HSSS in 81.3% (both sensitivity maximized strategy); the latter had one query less and the differences between three out of five dissimilar queries were due to the distance between two terms by using proximity operator. For example, “unlabel* indication*.af.” in MEDLINE HSSS was replaced by “(unlabel* adj3 indication*).af.” in EMBASE HSSS. Among 23 queries included in EMBASE HSSS (sensitivity- and precision-maximized), 11 queries were identical to the respective MEDLINE strategy. It also included a very long query (NOTed out “unlicensed”) and two more queries than MEDLINE.

We found that using an exclusion strategy was efficient to develop the optimized sensitive and precise strategy. It resulted from attempts to remove irrelevant studies whilst retaining sensitivity in two phases. The first one resulted from improving the retrieval by eliminating irrelevant content with search term “unlicensed”. In the second phase, we excluded studies on off-label use of medical devices, in particular stents, and veterinary medications from the selected set of queries, as it was the case in our prior study on developing a MEDLINE strategy [16]. It is likely that NOT operators impair sensitivity of a search [29,30] due to removing relevant as well as irrelevant records. Alternatively, there is a rather safe approach to use this operator. For example, the Cochrane HSSS (sensitivity maximizing) for identifying randomized trials in MEDLINE consisted of a query “animals [mh] NOT humans [mh]” which is combined by NOT to OR string of eight search terms [31]. Thus, this strategy excludes reports solely of animal studies, but retains reports indexed as human and animal, and neither human nor animal. Hence, we chose the search terms to combine with NOT if they were frequently used in the irrelevant records and rarely appeared in the relevant records only. Nonetheless, the sensitivity was slightly reduced as a consequence of excluding some relevant records. A recent study showed that NOTing out irrelevant content could improve retrieval of original studies on diagnosis, prognosis and etiology in MEDLINE, EMBASE, CINAHL and PsycINFO [32]. We also truncated short words like “use” or “out” within an exact sequence of words only. The truncated short terms are not generally recommended because they could retrieve many different words yielded low precision due to retrieving irrelevant records and in some database interfaces unexpected results may happen. Therefore, we carefully followed these short terms with an “exact” sequence of words to decrease its possible “side effects” associated with irrelevant records retrieval.

## Conclusion

We developed highly sensitive search strategies for OvidSP EMBASE to enhance the retrieval of studies on off-label drug use. Our study demonstrates that a comprehensive search for off-label drug use in OvidSP EMBASE can be much improved by highly sensitive search strategies instead of using simple search terms.

## Abbreviations

Ab: Abstract; ab,ti: Abstract or title; af: All searchable fields; EMBASE: Excerpta Medica Database; HSSS: Highly sensitive search strategy; mh: Medical Subject Heading (MeSH) term (‘exploded’); mp: Title, abstract, subject headings, heading word, drug trade name, original title, device manufacturer, drug manufacturer name; NNR: Number needed to read; sh: Medical Subject Headings; ti: Title.

## Competing interests

The authors declare that they have no competing interests.

## Authors’ contributions

HH had the idea. All authors contributed to the design of the study and construction of the study protocol. BM researched data and discussed the results with all authors. HH was responsible for statistical evaluations and interpretation of data. BM wrote the first drafts of the paper, which were critically revised by HH and MM. All authors read and approved the final manuscript.

## Pre-publication history

The pre-publication history for this paper can be accessed here:

http://www.biomedcentral.com/1471-2288/12/190/prepub

## Supplementary Material

Additional file 1 **Table S1.** Sensitivity, precision and number needed to read (NNR) of search queries in OvidSP EMBASE. Click here for file

Additional file 2 **Table S2.** Combinations of search queries with the best optimization of sensitivity and precision for detecting off-label drug use reports in OvidSP EMBASE. Click here for file

Additional file 3 OvidSP EMBASE Characteristics: Examples. Click here for file

## References

[B1] Alexander GC Gallagher SA Mascola A Moloney RM Stafford RS Increasing off-label use of antipsychotic medications in the United States, 1995-2008 Pharmacoepidemiol Drug Saf 2011 20 177 184 10.1002/pds.2082 21254289 PMC3069498

[B2] de Souza JA Advances in drug development: off-label drug utilization in oncology Clin Adv Hematol Oncol 2011 9 473 475 21841748

[B3] Lat I Micek S Janzen J Cohen H Olsen K Haas C Off-label medication use in adult critical care patients J Crit Care 2011 26 89 94 10.1016/j.jcrc.2010.06.012 20716478

[B4] Lee E Teschemaker AR Johann-Liang R Bazemore G Yoon M Shim KS Daniel M Pittman J Wutoh AK Off-label prescribing patterns of antidepressants in children and adolescents Pharmacoepidemiol Drug Saf 2012 21 137 144 10.1002/pds.2145 21538674

[B5] Logan AC Yank V Stafford RS Off-label use of recombinant factor VIIa in U.S. hospitals: analysis of hospital records Ann Intern Med 2011 154 516 522 21502649 10.7326/0003-4819-154-8-201104190-00002 PMC4011398

[B6] Ajuwon GA Use of the Internet for health information by physicians for patient care in a teaching hospital in Ibadan, Nigeria Biomed Digit Libr 2006 3 12 10.1186/1742-5581-3-12 17163991 PMC1764751

[B7] Sahapong S Manmart L Ayuvat D Potisat S Information use behavior of clinicians in evidence-based medicine process in Thailand J Med Assoc Thai 2009 92 435 441 19301740

[B8] Briggs K Crowlesmith I EMBASE—The excerpta medica database: Quick and comprehensive drug information Publish Res Q 1995 11 51 60 10.1007/BF02680446

[B9] University of York, NHS centre for reviews and dissemination Finding studies for systematic reviews: a resource list for researchers 2010 Centre for Reviews and Dissemination, University of York, York

[B10] Wong SS Wilczynski NL Haynes RB Comparison of top-performing search strategies for detecting clinically sound treatment studies and systematic reviews in MEDLINE and EMBASE J Med Libr Assoc 2006 94 451 455 17082841 PMC1629423

[B11] Bahaadinbeigy K Yogesan K Wootton R MEDLINE versus EMBASE and CINAHL for telemedicine searches Telemed J E Health 2010 16 916 919 10.1089/tmj.2010.0046 20925566

[B12] Kelly L St Pierre-Hansen N So many databases, such little clarity: Searching the literature for the topic aboriginal Can Fam Physician 2008 54 1572 1573 19005131 PMC2592335

[B13] Sampson M Barrowman NJ Moher D Klassen TP Pham B Platt R St John PD Viola R Raina P Should meta-analysts search Embase in addition to Medline? J Clin Epidemiol 2003 56 943 955 10.1016/S0895-4356(03)00110-0 14568625

[B14] Wilkins T Gillies RA Davies K EMBASE versus MEDLINE for family medicine searches: can MEDLINE searches find the forest or a tree? Can Fam Physician 2005 51 848 849 16926954 PMC1479531

[B15] Sampson M McGowan J Lefebvre C Moher D Grimshaw J PRESS: peer review of electronic search strategies 2008 Canadian Agency for Drugs and Technologies in Health, Ottawa

[B16] Mesgarpour B Müller M Herkner H Search strategies- identified reports on “off-label” drug use in MEDLINE J Clin Epidemiol 2012 65 827 834 10.1016/j.jclinepi.2012.01.020 22726764

[B17] Gaudio PA Ranibizumab for uveitic macular edema: why? Am J Ophthalmol 2009 148 179 180 10.1016/j.ajo.2009.04.019 19619717

[B18] Acharya NR Hong KC Lee SM Ranibizumab for refractory uveitis-related macular edema Am J Ophthalmol 2009 148 303 309 e302 10.1016/j.ajo.2009.03.028 19427988

[B19] Caron C Practice tips. Inserting the levonorgestrel intrauterine system: off-label use Can Fam Physician 2007 53 643 644 17872714 PMC1952592

[B20] Daskalaki I Spain CV Long SS Watson B Implementation of rotavirus immunization in Philadelphia, Pennsylvania: high levels of vaccine ineligibility and off-label use Pediatrics 2008 122 e33 e38 10.1542/peds.2007-2464 18595974

[B21] Konda C Rao AG Colchicine in dermatology Indian J Dermatol Venereol Leprol 2010 76 201 205 10.4103/0378-6323.60552 20228563

[B22] de Jong J van den Berg PB Visser ST de Vries TW de Jong-van den Berg LT Antibiotic usage, dosage and course length in children between 0 and 4 years Pharmacoepidemiol Drug Saf 2009 18 S213 S214

[B23] de Jong J van den Berg PB Visser ST de Vries TW de Jong-van den Berg LT Antibiotic usage, dosage and course length in children between 0 and 4 years Acta Paediatr 2009 98 1142 1148 10.1111/j.1651-2227.2009.01309.x 19397542

[B24] Matar HE Almerie MQ Adams CE Essali A Publications indexed in Medline and Embase originating from the Syrian Arab Republic: a survey East Mediterr Health J 2009 15 648 652 19731781

[B25] Excerpta Medica Database (EMBASE) Release fact sheet (summer 2009) 2009 Elsevier B.V, The Netherlands Available from http://www.embase.com/info/UserFiles/Aug1ReleaseFactSheet_0.pdf

[B26] Chalumeau M Treluyer JM Salanave B Assathiany R Cheron G Crocheton N Rougeron C Mares M Breart G Pons G Off label and unlicensed drug use among French office based paediatricians Arch Dis Child 2000 83 502 505 10.1136/adc.83.6.502 11087286 PMC1718582

[B27] Mukattash T Hawwa AF Trew K McElnay JC Healthcare professional experiences and attitudes on unlicensed/off-label paediatric prescribing and paediatric clinical trials Eur J Clin Pharmacol 2011 67 449 461 10.1007/s00228-010-0978-z 21243345

[B28] Glanville J Kaunelis D Mensinkai S How well do search filters perform in identifying economic evaluations in MEDLINE and EMBASE Int J Technol Assess Health Care 2009 25 522 529 10.1017/S0266462309990523 19845982

[B29] Deacon P Smith JB Tow S Using metadata to create navigation paths in the HealthInsite Internet gateway Health Info Libr J 2001 18 20 29 10.1046/j.1365-2532.2001.00319.x 11260289

[B30] Jenuwine ES Floyd JA Comparison of medical subject headings and text-word searches in MEDLINE to retrieve studies on sleep in healthy individuals J Med Libr Assoc 2004 92 349 353 15243641 PMC442177

[B31] Lefebvre C Manheimer E Glanville J Higgins JPT, Green S Chapter 6: Searching for studies Cochrane handbook for systematic reviews of interventions version 5.1.0 (Updated march 2011) 2011 Available from http://www.cochrane-handbook.org

[B32] Wilczynski NL McKibbon KA Haynes RB Search filter precision can be improved by NOTing out irrelevant content AMIA Annu Symp Proc 2011 2011 1506 1513 22195215 PMC3243169

