# Supplementary material for: Search strategies to identify reports on “off-label” drug use in EMBASE
Source: BMC Med Res Methodol. 2012 Dec 29;12:190. doi: 10.1186/1471-2288-12-190 (PMC3543848; doi:10.1186/1471-2288-12-190)
Supplement: Additional file 1 — Table S1. Sensitivity, precision and number needed to read (NNR) of search queries in OvidSP EMBASE. [file 1471-2288-12-190-S1.pdf]

**Table S1- Sensitivity, precision and number needed to read (NNR) of search queries in OvidSP EMBASE.** Text word (TW) field in OvidSP EMBASE includes only title and abstract information. Then, we presented our search queries only with “ab,ti”.

| Code | Search query | Number of relevant records retrieved | Number of records retrieved | Sensitivity/ Full set (%) | Sensitivity/ EMBASE set (%) | Precision (%) | NNR |
|---|---|---|---|---|---|---|---|
| **1** | off label*.af. | 3150 | 3577 | 77.5 | 81.9 | 88.1 | 1.1 |
| **2** | "off label*".ti. | 728 | 819 | 17.9 | 18.9 | 88.9 | 1.1 |
| **3** | "off label*".ab. | 1882 | 2224 | 46.3 | 48.9 | 84.6 | 1.2 |
| **4** | "off label*".ab,ti. | 2306 | 2696 | 56.7 | 60.0 | 85.5 | 1.2 |
| **5** | off label*.mp. | 3128 | 3555 | 76.9 | 81.3 | 88.0 | 1.1 |
| **6** | (off adj1 label*).mp. | 3130 | 3567 | 77.0 | 81.4 | 87.7 | 1.1 |
| **7** | (off adj2 label*).mp. | 3131 | 3589 | 77.0 | 81.4 | 87.2 | 1.1 |
| **8** | off label.af. | 3119 | 3542 | 76.7 | 81.1 | 88.1 | 1.1 |
| **9** | off label.ti. | 726 | 817 | 17.9 | 18.9 | 88.9 | 1.1 |
| **10** | off label.ab. | 1870 | 2208 | 46.0 | 48.6 | 84.7 | 1.2 |
| **11** | off label.ab,ti. | 2293 | 2679 | 56.4 | 59.6 | 85.6 | 1.2 |
| **12** | off label.mp. | 3117 | 3540 | 76.6 | 81.0 | 88.1 | 1.1 |
| **13** | (off adj1 label).mp. | 3119 | 3544 | 76.7 | 81.1 | 88.0 | 1.1 |
| **14** | (off adj2 label).mp. | 3119 | 3547 | 76.7 | 81.1 | 87.9 | 1.1 |
| **15** | offlabel*.af. | 23 | 27 | 0.6 | 0.6 | 85.2 | 1.2 |
| **16** | "off label drug use".sh. | 1498 | 1594 | 36.8 | 38.9 | 94.0 | 1.1 |
| **17** | "off label drug use".af. | 1551 | 1647 | 38.1 | 40.3 | 94.2 | 1.1 |
| **18** | "off label drug us*".af. | 1580 | 1677 | 38.8 | 41.1 | 94.2 | 1.1 |
| **19** | (label adj1 us*).af. | 1146 | 1674 | 28.2 | 29.8 | 68.5 | 1.5 |
| **20** | (drug adj1 label adj1 us*).af. | 1581 | 1710 | 38.9 | 41.1 | 92.5 | 1.1 |
| **21** | (drug adj2 label adj2 us*).af. | 1587 | 1719 | 39.0 | 41.3 | 92.3 | 1.1 |
| **22** | (label adj1 indication*).af. | 159 | 247 | 3.9 | 4.1 | 64.4 | 1.6 |
| **23** | (label adj2 indication*).af. | 178 | 276 | 4.4 | 4.6 | 64.5 | 1.6 |
| **24** | (label adj3 indication*).af. | 206 | 310 | 5.1 | 5.4 | 66.5 | 1.5 |
| **25** | (without label).af. | 0 | 14 | 0.0 | 0.0 | 0.0 | - |
| **26** | (out of label).af. | 4 | 18 | 0.1 | 0.1 | 22.2 | 4.5 |
| **27** | (out of label*).af. | 4 | 82 | 0.1 | 0.1 | 4.9 | 20.5 |
| **28** | unlabel* us*.af. | 32 | 35 | 0.8 | 0.8 | 91.4 | 1.1 |
| **29** | (unlabel* adj1 us*).af. | 32 | 221 | 0.8 | 0.8 | 14.5 | 6.9 |
| **30** | unlabel* indication*.af. | 16 | 16 | 0.4 | 0.4 | 100.0 | 1.0 |
| **31** | (unlabel* adj3 indication*).af. | 19 | 19 | 0.5 | 0.5 | 100.0 | 1.0 |
| **32** | (improper adj1 indication*).af. | 4 | 8 | 0.1 | 0.1 | 50.0 | 2.0 |

| | | | | | | | |
|---|---|---|---|---|---|---|---|
| **33** | without proper indication*.af. | 3 | 11 | 0.1 | 0.1 | 27.3 | 3.7 |
| **34** | registered indication*.af. | 24 | 39 | 0.6 | 0.6 | 61.5 | 1.6 |
| **35** | (inappropriate indication*).af. | 29 | 144 | 0.7 | 0.8 | 20.1 | 5.0 |
| **36** | (inappropriate adj5 indication adj2 us*).af. | 4 | 9 | 0.1 | 0.1 | 44.4 | 2.3 |
| **37** | (appropriate indication adj3 us*).af. | 6 | 11 | 0.1 | 0.2 | 54.5 | 1.8 |
| **38** | (prescrib* adj2 inappropriate indication*).af. | 2 | 3 | 0.0 | 0.1 | 66.7 | 1.5 |
| **39** | ((appropriate* adj3 prescri*) and indication).af. | 48 | 127 | 1.2 | 1.2 | 37.8 | 2.6 |
| **40** | no* appropriate indication*.af. | 4 | 11 | 0.1 | 0.1 | 36.4 | 2.8 |
| **41** | ((inappropriate us* and indication) not (antibiotic* or antimicrobial)).af. | 54 | 144 | 1.3 | 1.4 | 37.5 | 2.7 |
| **42** | unapprove*.af. | 222 | 379 | 5.5 | 5.8 | 58.6 | 1.7 |
| **43** | unapprove* us*.af. | 65 | 72 | 1.6 | 1.7 | 90.3 | 1.1 |
| **44** | (unapprove* adj2 us*).af. | 88 | 120 | 2.2 | 2.3 | 73.3 | 1.4 |
| **45** | (unapprove* adj1 prescription).af. | 2 | 3 | 0.0 | 0.1 | 66.7 | 1.5 |
| **46** | (unapprove* adj5 prescription).af. | 8 | 10 | 0.2 | 0.2 | 80.0 | 1.3 |
| **47** | (unapprove* adj indication*).af. | 34 | 39 | 0.8 | 0.9 | 87.2 | 1.1 |
| **48** | (unapprove* adj2 indication*).af. | 39 | 45 | 1.0 | 1.0 | 86.7 | 1.2 |
| **49** | (unapprove* adj3 indication*).af. | 63 | 70 | 1.5 | 1.6 | 90.0 | 1.1 |
| **50** | (unapprove* adj5 indication*).af. | 77 | 85 | 1.9 | 2.0 | 90.6 | 1.1 |
| **51** | (unapprove* adj1 drug*).af. | 33 | 59 | 0.8 | 0.9 | 55.9 | 1.8 |
| **52** | (unapprove* adj2 drug*).af. | 44 | 80 | 1.1 | 1.1 | 55.0 | 1.8 |
| **53** | nonapprove*.af. | 26 | 58 | 0.6 | 0.7 | 44.8 | 2.2 |
| **54** | non fda approve*.af. | 17 | 35 | 0.4 | 0.4 | 48.6 | 2.1 |
| **55** | off li?en?e.af. | 75 | 85 | 1.8 | 2.0 | 88.2 | 1.1 |
| **56** | off li?en?e*.af. | 76 | 93 | 1.9 | 2.0 | 81.7 | 1.2 |
| **57** | unlicen?e*.af. | 277 | 860 | 6.8 | 7.2 | 32.2 | 3.1 |
| **58** | unlicense*.af. | 274 | 853 | 6.7 | 7.1 | 32.1 | 3.1 |
| **59** | (unlicen?e* adj3 indication*).af. | 39 | 41 | 1.0 | 1.0 | 95.1 | 1.1 |
| **60** | (unlicen?e* adj3 treatment*).af. | 10 | 13 | 0.2 | 0.3 | 76.9 | 1.3 |
| **61** | (unlicense* adj1 therapy).af. | 1 | 2 | 0.0 | 0.0 | 50.0 | 2.0 |
| **62** | unlicensed manner.af. | 7 | 7 | 0.2 | 0.2 | 100.0 | 1.0 |
| **63** | (unlicensed not (unlicensed aide* or unlicensed assist* or unlicensed car* or (Unlicensed adj2 heal*)or unlicensed home* or (killer adj2 cell*) or (unlicensed adj2 individual*) or (unlicensed adj2 nurs*) or (unlicensed adj4 practi*) or (unlicensed adj2 physician*) or (unlicensed adj2 operat*) or (unlicensed adj2 person*) or unlicensed profession* or unlicensed rid* or (unlicensed adj3 staff*) or unlicensed therapist* or (unlicensed | 262 | 422 | 6.4 | 6.8 | 62.1 | 1.6 |

| | | | | | | | |
|---|---|---|---|---|---|---|---|
| | adj5 vaccine*) or unlicensed vendor* or (unlicensed adj2 work*) or (unlicensed adj2 employe*) or device*or dentist*or driver or driving or herbal or medical graduate* or motor* or pesticide*or premis*or prostitute*or restaurant*or veterinary or worker*)).af. | | | | | | |
| **64** | (outside licen?e*).af. | 5 | 7 | 0.1 | 0.1 | 71.4 | 1.4 |
| **65** | (outside adj2 licen?e*).af. | 27 | 36 | 0.7 | 0.7 | 75.0 | 1.3 |
| **66** | (outside adj3 licen?e*).af. | 43 | 55 | 1.1 | 1.1 | 78.2 | 1.3 |
| **67** | (out* adj4 licen?ed indication*).af. | 22 | 25 | 0.5 | 0.6 | 88.0 | 1.1 |
| **68** | (be???d* adj2 licen?ed indication*).af. | 5 | 6 | 0.1 | 0.1 | 83.3 | 1.2 |
| **69** | ((no* licen?ed for adj3 use*) not now licen?ed).af. | 70 | 104 | 1.7 | 1.8 | 67.3 | 1.5 |
| **70** | ((no* licen?ed for adj3 indication*) not now licen?ed).af. | 15 | 17 | 0.4 | 0.4 | 88.2 | 1.1 |
| **71** | (us* without adj2 indication*).af. | 5 | 11 | 0.1 | 0.1 | 45.5 | 2.2 |
| **72** | (drug* without adj2 indication*).af. | 2 | 5 | 0.0 | 0.1 | 40.0 | 2.5 |
| **73** | (medication adj2 without adj2 indication*).af. | 1 | 2 | 0.0 | 0.0 | 50.0 | 2.0 |
| **74** | (non evidence base* us*).af. | 4 | 6 | 0.1 | 0.1 | 66.7 | 1.5 |
| **75** | (prescri* outside adj4 guideline*).af. | 5 | 6 | 0.1 | 0.1 | 83.3 | 1.2 |
| **76** | labeled indication*.af. | 31 | 53 | 0.8 | 0.8 | 58.5 | 1.7 |
| **77** | label off.af. | 10 | 12 | 0.2 | 0.3 | 83.3 | 1.2 |

“af” indicates all searchable fields; “ti”, title; “ab”, abstract; “ab,ti”, abstract or title; “mp”, title, abstract, subject headings, heading word, drug trade name, original title, device manufacturer or drug manufacturer name; “sh”, Medical Subject Headings.
