# Supplementary material for: Search strategies to identify reports on “off-label” drug use in EMBASE
Source: BMC Med Res Methodol. 2012 Dec 29;12:190. doi: 10.1186/1471-2288-12-190 (PMC3543848; doi:10.1186/1471-2288-12-190)
Supplement: Additional file 2 — Table S2. Combinations of search queries with the best optimization of sensitivity and precision for detecting off-label drug use reports in OvidSP EMBASE. [file 1471-2288-12-190-S2.pdf]

Table S2- Combinations of search queries with the best optimization of sensitivity and precision for detecting off-label drug use reports in OvidSP EMBASE

Search strategy	# of search queries	# of relevant records retrieved	# of records retrieved	Sensitivity/ full set (%)	Precision (%)
(or/1,15,28,31,32,34,36,37,44,50,52-55,63,66-76) not (stent* or veterinar*).af.	26	3641	4173	89.53	87.25
(or/1,15,28,31,32,34,36,37,44,50,52-55,63,66-71,73-76) not (stent* or veterinar*).af.	25	3640	4170	89.50	87.29
(or/1,15,28,31,32,34,36,37,44,50,52-55,63,66-71,74-76) not (stent* or veterinar*).af.	24	3639	4168	89.48	87.31
(or/1,15,28,31,34,36,37,44,50,52-55,63,66-71,73-76) not (stent* or veterinar*).af.	24	3638	4164	89.45	87.37
(or/1,15,28,31,34,36,37,44,50,52-55,63,66-71,74-76) not (stent* or veterinar*).af.	23	3637	4162	89.43	87.39
(or/1,15,28,31,34,36,37,44,50,52-55,63,66,68-71,73-76) not (stent* or veterinar*).af.	23	3636	4160	89.40	87.40
(or/1,15,28,31,34,36,37,44,50,52-55,63,66,68-71,74-76) not (stent* or veterinar*).af. †	22	3635	4158	89.38	87.42
(or/1,13,15,28,31-34,36,37,44,50,52-55,63,66-72,74,75) not (stent* or veterinar*).af.	26	3634	4158	89.35	87.40
(or/1,15,28,31,34,36,37,44,50,52-55,63,66-71,75-76) not (stent* or veterinar*).af.	22	3634	4158	89.35	87.40
(or/1,13,15,28,31-34,36,37,44,50,52-55,63,66-71,74,75) not (stent* or veterinar*).af.	25	3633	4155	89.33	87.44
(or/1,13,15,28,31,32,34,36,37,44,50,52-55,63,66-75) not (stent* or veterinar*).af.	26	3632	4149	89.30	87.54
(or/1,13,15,28,31,32,34,36,37,44,50,52-55,63,66-71,73-75) not (stent* or veterinar*).af.	25	3631	4146	89.28	87.58
(or/1,13,15,28,31,32,34,36,37,44,50,52-55,63,66-71,74,75) not (stent* or veterinar*).af.	24	3630	4144	89.25	87.60
(or/1,13,15,28,31,34,36,37,44,50,52-55,63,66-71,73-75) not (stent* or veterinar*).af.	24	3629	4140	89.23	87.66
(or/1,13,15,28,31,34,36,37,44,50,52-55,63,66-71,74,75) not (stent* or veterinar*).af.	23	3628	4138	89.21	87.68

† See the expansion of search strategy in table 3
