# Supplementary material for: Search strategies to identify reports on “off-label” drug use in EMBASE
Source: BMC Med Res Methodol. 2012 Dec 29;12:190. doi: 10.1186/1471-2288-12-190 (PMC3543848; doi:10.1186/1471-2288-12-190)
Supplement: Additional file 3 — OvidSP EMBASE Characteristics: Examples. [file 1471-2288-12-190-S3.pdf]

OvidSP EMBASE Characteristics: Examples *[update: October 3, 2012]*

Example 1: Duplicate records of a study: two accession numbers with similar entry week

Wolters Kluwer Health | OvidSP

My Account | Support & Training | Help | Logoff

Search | Journals | Books | My Workspace

Keep Selected

Search Results

1.

- Find Similar
- Find Citing Articles
- ubmed findit

Accession Number	17872714
Authors	Caron C.
Institution	(Caron) Department of Family Medicine, University of Ottawa, Ontario.
Correspondence Address	C. Caron, Department of Family Medicine, University of Ottawa, Ontario.
Country of Publication	Canada
Title	Practice tips. Inserting the levonorgestrel intrauterine system: off-label use.
Source	Canadian family physician Medecin de famille canadien. 53 (4) (pp 643-644), 2007. Date of Publication: Apr 2007.
Subject Headings	article<br>Canada<br>*contraception<br>female<br>follow up<br>general practitioner<br>human<br>*intrauterine contraceptive device<br>methodology<br>risk assessment<br>*levonorgestrel / ad [Drug Administration]
CAS Registry Numbers	797-63-7 (levonorgestrel)
Electronic ISSN	1715-5258
Language	English
Publication Type	Journal: Article
Entry Week	200700
Date Delivered	20071023
Year of Publication	2007
Copyright	MEDLINE is the source for the citation and abstract of this record.

+ My Projects

Search Results

Keep Selected

English | Français | Deutsch | 日本語 | 繁體中文 | Español | 简体中文 | 한국어

Copyright (c) 2000-2012 Ovid Technologies, Inc.

Terms of Use | Support & Training | About Us | Contact Us

Version: OvidSP_UI03.06.00.122, SourceID 56772

Wolters Kluwer Health | OvidSP

My Account | Support & Training | Help | Logoff

Search | Journals | Books | My Workspace

Keep Selected

Search Results

1.

- Find Similar
- Find Citing Articles
- ubmed findit

Accession Number	2007205136
Authors	Caron C.
Institution	(Caron) Department of Family Medicine, University of Ottawa, Ont., Canada
Correspondence Address	C. Caron, Department of Family Medicine, University of Ottawa, Ont., Canada
Country of Publication	Canada
Title	Inserting the levonorgestrel intrauterine system: Off-label use.
Source	Canadian Family Physician. 53 (4) (pp 643-644), 2007. Date of Publication: April 2007.
Publisher	College of Family Physicians of Canada
URL	http://www.cfpc.ca/cfp/2007/Apr/...
Subject Headings	amenorrhea<br>article<br>drug cost<br>drug delivery system<br>drug efficacy<br>dysmenorrhea / dt [Drug Therapy]<br>endometrial disease / co [Complication]<br>endometriosis / dt [Drug Therapy]<br>endometrium thinning / co [Complication]<br>human<br>menorrhagia / dt [Drug Therapy]<br>menstrual irregularity / si [Side Effect]<br>menstruation<br>muscle cramp / dt [Drug Therapy]<br>muscle cramp / pc [Prevention]<br>off label drug use<br>premenstrual syndrome / si [Side Effect]<br>uterus cramp / dt [Drug Therapy]<br>uterus perforation / co [Complication]<br>ibuprofen / dt [Drug Therapy]<br>ibuprofen / po [Oral Drug Administration]<br>*levonorgestrel / ae [Adverse Drug Reaction]<br>*levonorgestrel / po [Oral Drug Administration]<br>*levonorgestrel / pr [Pharmaceutics]<br>*levonorgestrel / pe [Pharmacoeconomics]
Number of References	2
Embase Section Headings	Biophysics, Bioengineering and Medical Instrumentation [27]<br>Health Policy, Economics and Management [36]<br>Drug Literature Index [37]<br>Adverse Reactions Titles [38]<br>Pharmacy [39]
Drug Trade Names and Manufacturers	mirena
Device Trade Names and Manufacturers	loestrin, mirena
CAS Registry Numbers	15687-27-1 (ibuprofen); 797-63-7 (levonorgestrel)
ISSN	0008-350X
CODEN	CFPHA
Language	English
Publication Type	Journal: Article
Entry Week	200700
Date Delivered	20070509
Year of Publication	2007
Copyright	Copyright 2009 Elsevier B.V., All rights reserved.

+ My Projects

Search Results

Example 2- Duplicate records of a study: two accession numbers with two entry weeks

Wolters Kluwer Health | OvidSP

My Account | Support & Training | Help | Logoff

Search | Journals | Books | My Workspace

Keep Selected

Search Results

1. ☐

- Abstract Reference
- Find Similar
- Find Citing Articles
- ubmed findit

Accession Number	18595974
Authors	Daskalaki I. Spain C.V. Long S.S. Watson B.
Institution	(Daskalaki, Spain, Long, Watson) Department of Pediatrics, St Christopher's Hospital for Children, Philadelphia, PA 19134, USA.
Correspondence Address	I. Daskalaki, Department of Pediatrics, St Christopher's Hospital for Children, Philadelphia, PA 19134, USA.
Country of Publication	United States
Title	Implementation of rotavirus immunization in Philadelphia, Pennsylvania: high levels of vaccine ineligibility and off-label use.
Source	Pediatrics. 122 (1) (pp e33-38), 2008. Date of Publication: Jul 2008.
Subject Headings	age distribution<br>article<br>human<br>*immunization<br>infant<br>preschool child<br>public health<br>United States<br>utilization review<br>diphtheria pertussis tetanus vaccine<br>*Rotavirus vaccine / dt [Drug Therapy]
Abstract	OBJECTIVE: Our goal was to predict, using delayed diphtheria-tetanus-acellular pertussis vaccination as an indicator, whether the current narrowly defined age limits for pentavalent rotavirus vaccine exclude a substantial proportion of children from complete immunization against rotavirus and to assess adherence of providers to recommended age limits by examining the first 6 months of use of pentavalent rotavirus vaccine in Philadelphia, Pennsylvania. PATIENTS AND METHODS: Data from a computerized children's immunization registry in Philadelphia were analyzed. Demographics and age at immunization with first 3 diphtheria-tetanus-acellular pertussis doses were examined from 2001 to 2005. Similar characteristics were evaluated for children who received pentavalent rotavirus vaccine doses during the first 6 months of its availability (August 2006 through January 2007). RESULTS: During the 5-year period, 24 403 of 103 967 recipients of first diphtheria-tetanus-acellular pertussis vaccine were >12 weeks of age; only 56 411 of 79 564 first diphtheria-tetanus-acellular pertussis recipients <or=12 weeks of age received the first 3 doses at ages that they could have completed the pentavalent rotavirus vaccine series if vaccines were given at the same visit. Children using public providers were more likely to have delayed immunization. During the first 6 months of pentavalent rotavirus vaccine implementation, 5566 pentavalent rotavirus vaccine doses were recorded in the Kids Immunization Database/Tracking System: 3912 first doses, 1419 second doses, and 235 third doses. Of 3912 first-dose pentavalent rotavirus vaccine recipients, 770 were >12 weeks of age. Hospital-based providers were less likely to administer pentavalent rotavirus vaccine off-label. CONCLUSIONS: With the current level of vaccine implementation and current pentavalent rotavirus vaccine recommendations for series initiation, a substantial proportion of children are expected to be excluded from receiving any pentavalent rotavirus vaccine or completing the series. In the first 6 months of availability, pentavalent rotavirus vaccine frequently was used off-label for age, underscoring the importance of education of immunization providers. Current outreach programs for finding 10-month-old toddlers delinquent for immunizations will not improve the possibility of protection against rotavirus.
Electronic ISSN	1098-4275
DOI	http://dx.doi.org/10.1542/peds.2...
Language	English
Publication Type	Journal: Article
Entry Week	200800
Date Delivered	20080804
Year of Publication	2008
Copyright	MEDLINE is the source for the citation and abstract of this record.

+ My Projects

Search Results

Wolters Kluwer Health | OvidSP
My Account | Support & Training | Help | Logoff
Search Journals Books My Workspace
Keep Selected
Search Results

1.

Abstract Reference
Find Similar
Find Citing Articles
ubmed findit

Accession Number 2009161626

Authors Daskalaki I. Spai C.V. Long S.S. Watson B.

Institution (Daskalaki, Long) Section of Infectious Diseases, St Christopher's Hospital for Children, Philadelphia, PA
(Daskalaki, Long) Department of Pediatrics, Drexel University, College of Medicine, Philadelphia, PA
(Spai, Watson) Division of Disease Control, Philadelphia Department of Public Health, Philadelphia, PA
(Daskalaki) St Christopher's Hospital for Children, Department of Pediatrics, Erie Avenue at Front Street, Philadelphia, PA 19134

Correspondence Address I. Daskalaki, St Christopher's Hospital for Children, Department of Pediatrics, Erie Avenue at Front Street, Philadelphia, PA 19134. E-mail: Irini.daskalaki@drexelmed.edu

Country of Publication United States

Title Implementation of rotavirus immunization in Philadelphia, Pennsylvania: High levels of vaccine ineligibility and off-label use.

Source Pediatrics. 122 (1) (pp e33-e38), 2008. Date of Publication: July 2008.

Publisher American Academy of Pediatrics (141 Northwest Point Blvd, P.O. Box 927, Elk Grove Village IL 60007-1098, United States)

Keyword Immunization, Off-label, Rotavirus, Timeliness

URL http://pediatrics.aappublication...

Subject Headings
article
health care personnel
health education
health program
hospital care
human
*immunization
major clinical study
newborn
*off label drug use
patient compliance
priority journal
*Rotavirus
treatment duration
United States
*virus infection / dt [Drug Therapy]
*virus infection / pc [Prevention]
*diphtheria pertussis tetanus vaccine / dt [Drug Therapy]
*Rotavirus vaccine / dt [Drug Therapy]

Abstract Objective. Our goal was to predict, using delayed diphtheria-tetanus- acellular pertussis vaccination as an indicator, whether the current narrowly defined age limits for pentavalent rotavirus vaccine exclude a substantial proportion of children from complete immunization against rotavirus and to assess adherence of providers to recommended age limits by examining the first 6 months of use of pentavalent rotavirus vaccine in Philadelphia, Pennsylvania. PATIENTS AND METHODS. Data from a computerized children's immunization registry in Philadelphia were analyzed. Demographics and age at immunization with first 3 diphtheria-tetanus-acellular pertussis doses were examined from 2001 to 2005. Similar characteristics were evaluated for children who received pentavalent rotavirus vaccine doses during the first 6 months of its availability (August 2006 through January 2007). RESULTS. During the 5-year period, 24 403 of 103 967 recipients of first diphtheria- tetanus-acellular pertussis vaccine were >12 weeks of age;only 56 411 of 79 564 first diphtheria-tetanus-acellular pertussis recipients <=12 weeks of age received the first 3 doses at ages that they could have completed the pentavalent rotavirus vaccine series if vaccines were given at the same visit. Children using public providers were more likely to have delayed immunization. During the first 6 months of pentavalent rotavirus vaccine implementation, 5566 pentavalent rotavirus vaccine doses were recorded in the Kids Immunization Database/Tracking System: 3912 first doses, 1419 second doses, and 235 third doses. Of 3912 first-dose pentavalent rotavirus vaccine recipients, 770 were >12 weeks of age. Hospital-based providers were less likely to administer pentavalent rotavirus vaccine off-label. CONCLUSIONS. With the current level of vaccine implementation and current pentavalent rotavirus vaccine recommendations for series initiation, a substantial proportion of children are expected to be excluded from receiving any pentavalent rotavirus vaccine or completing the series. In the first 6 months of availability, pentavalent rotavirus vaccine frequently was used off-label for age, underscoring the importance of education of immunization providers. Current outreach programs for finding 10-month-old toddlers delinquent for immunizations will not improve the possibility of protection against rotavirus. Copyright 2008 by the American Academy of Pediatrics.

Number of References 33

Embase Section Headings
Pediatrics and Pediatric Surgery [7]
Public Health, Social Medicine and Epidemiology [17]
Immunology, Serology and Transplantation [26]
Drug Literature Index [37]

ISSN 0031-4005

Electronic ISSN 1098-4275

DOI http://dx.doi.org/10.1542/peds.2...

CODEN PEDIA

Language English

Summary Language English

Publication Type Journal: Article

Entry Week 200900

Date Delivered 20090427

Year of Publication 2008

Copyright Copyright 2011 Elsevier B.V., All rights reserved.

+ My Projects

Search Results

Example 3- Different records for one study: two different titles with different indexing

Wolters Kluwer Health | OvidSP

My Account | Support & Training | Help | Logoff

Search | Journals | Books | My Workspace

Keep Selected

1 GO | Search Results | Next

1.

- Find Similar
- Find Citing Articles
- ubmed findit

Accession Number	20228563
Authors	Konda C. Rao A.G.
Institution	(Konda) St. Theresa's Hospital, Sanathnagar, Hyderabad-500018, India.
Correspondence Address	C. Konda, St. Theresa's Hospital, Sanathnagar, Hyderabad-500018, India.
Country of Publication	India
Title	Colchicine in dermatology.
Source	Indian journal of dermatology, venereology and leprology. 76 (2) (pp 201-205), 2010. Date of Publication: 2010 Mar-Apr.
Subject Headings	animal<br>*autoimmune disease / dt [Drug Therapy]<br>*dermatology<br>human<br>immunology<br>methodology<br>pathology<br>review<br>*skin disease / dt [Drug Therapy]<br>*colchicine / ad [Drug Administration]
Number of References	55
CAS Registry Numbers	64-86-8 (colchicine)
Electronic ISSN	0973-3922
Language	English
Publication Type	Journal: Review
Entry Week	201000
Date Delivered	20100929
Year of Publication	2010
Copyright	MEDLINE is the source for the citation and abstract of this record.

+ My Projects

1 GO | Search Results | Next

Keep Selected

English | Français | Deutsch | 日本語 | 繁體中文 | Español | 简体中文 | 한국어

Copyright (c) 2000-2012 Ovid Technologies, Inc.

Terms of Use | Support & Training | About Us | Contact Us

Version: OvidSP_UI03.06.00.122, SourceID 56772

5.

Find Similar
ubmed findit

Accession Number 2010185181

Country of Publication India

Title Dosages and administration.

Source Indian Journal of Dermatology, Venereology and Leprology. 76 (2) (pp 202-205), 2010. Date of Publication: 01 Mar 2010.

Publisher Medknow Publications and Media Pvt. Ltd (B9, Kanara Business Centre, off Link Road, Ghatkopar (E), Mumbai 400 075, India)

Subject Headings

acute febrile neutrophilic dermatosis / dt [Drug Therapy]
alopecia / co [Complication]
alopecia / si [Side Effect]
amyloidosis / dt [Drug Therapy]
antiinflammatory activity
aphthous stomatitis / dt [Drug Therapy]
azoospermia / co [Complication]
azoospermia / si [Side Effect]
Behcet disease / dt [Drug Therapy]
bone marrow suppression / co [Complication]
bone marrow suppression / si [Side Effect]
bullous skin disease / dt [Drug Therapy]
clinical practice
drug contraindication
drug dose reduction
drug effect
drug indication
drug information
drug mechanism
drug monitoring
drug overdose / co [Complication]
drug structure
drug withdrawal
familial Mediterranean fever / dt [Drug Therapy]
gastrointestinal toxicity / co [Complication]
gastrointestinal toxicity / si [Side Effect]
*gout / dt [Drug Therapy]
*gout / pc [Prevention]
human
leukopenia / co [Complication]
leukopenia / dt [Drug Therapy]
leukopenia / si [Side Effect]
malabsorption / si [Side Effect]
megaloblastic anemia / si [Side Effect]
myopathy / si [Side Effect]
neuropathy / si [Side Effect]
off label drug use
patient monitoring
porphyria cutanea tarda / co [Complication]
porphyria cutanea tarda / si [Side Effect]
practice guideline
pregnancy
*psoriasis / dt [Drug Therapy]
recommended drug dose
review
risk
scleroderma / dt [Drug Therapy]
*skin disease / dt [Drug Therapy]
skin toxicity / co [Complication]
skin toxicity / si [Side Effect]
steatorrhea / si [Side Effect]
teratogenicity
toxic epidermal necrolysis / co [Complication]
toxic epidermal necrolysis / si [Side Effect]
urticaria / co [Complication]
urticaria / si [Side Effect]
vasculitis / dt [Drug Therapy]
allopurinol / dt [Drug Therapy]
antiinflammatory agent / dt [Drug Therapy]
*colchicine / ae [Adverse Drug Reaction]
*colchicine / ad [Drug Administration]
*colchicine / an [Drug Analysis]
*colchicine / cr [Drug Concentration]
*colchicine / do [Drug Dose]
*colchicine / it [Drug Interaction]
*colchicine / dt [Drug Therapy]
*colchicine / to [Drug Toxicity]
*colchicine / iv [Intravenous Drug Administration]
*colchicine / po [Oral Drug Administration]
*colchicine / pr [Pharmaceutics]
*colchicine / pk [Pharmacokinetics]
*colchicine / pd [Pharmacology]
cyanocobalamin / ae [Adverse Drug Reaction]
*colchicine / po [Oral Drug Administration]
*colchicine / pr [Pharmaceutics]
*colchicine / pk [Pharmacokinetics]
*colchicine / pd [Pharmacology]
cyanocobalamin / ae [Adverse Drug Reaction]
cyanocobalamin / it [Drug Interaction]
cyanocobalamin / pk [Pharmacokinetics]
cyclosporin / cr [Drug Concentration]
cyclosporin / it [Drug Interaction]
cyclosporin / pk [Pharmacokinetics]
granulocyte colony stimulating factor / dt [Drug Therapy]
immunosuppressive agent / dt [Drug Therapy]
macrolide / it [Drug Interaction]
simvastatin / ae [Adverse Drug Reaction]
simvastatin / it [Drug Interaction]
verapamil / cr [Drug Concentration]
verapamil / it [Drug Interaction]
verapamil / pk [Pharmacokinetics]

Number of References 55

Embase Section Headings

Dermatology and Venereology [13]
Clinical and Experimental Pharmacology [30]
Drug Literature Index [37]
Adverse Reactions Titles [38]
Pharmacy [39]

CAS Registry Numbers 315-30-0 (allopurinol); 64-86-8 (colchicine); 53570-76-6 (cyanocobalamin); 68-19-9 (cyanocobalamin); 8064-09-3 (cyanocobalamin); 79217-60-0 (cyclosporin); 79902-63-9 (simvastatin); 152-11-4 (verapamil); 52-53-9 (verapamil)

ISSN 0378-6323

Electronic ISSN 0973-3922

CODEN IJDLD

Language English

Publication Type Journal: Review

Entry Week 201000

Date Delivered 20100423

Year of Publication 2010

Copyright Copyright 2011 Elsevier B.V., All rights reserved.

Example 4- Different indexing for one study published as a conference abstract and an article

Wolters Kluwer Health | OvidSP

My Account | Support & Training | Help | Logoff

Search Journals Books My Workspace

Keep Selected

2 GO ≫ ◂ Previous | Search Results

- Abstract Reference
- Find Similar
- Find Citing Articles
- ubmed findit

2.

Accession Number	70069339
Authors	De Jong J. Van Den Berg P.B. Visser S.T. De Vries T.W. De Jong-Van Den Berg L.T.W.
Institution	(De Jong, Van Den Berg, Visser, De Jong-Van Den Berg) Pharmacoepidemiology and Pharmacoeconomics, University of Groningen, Groningen, Netherlands<br>(De Vries) Pediatrics, Medical Centre Leeuwarden, Leeuwarden, Netherlands
Correspondence Address	J. De Jong, Pharmacoepidemiology and Pharmacoeconomics, University of Groningen, Groningen, Netherlands
Title	Antibiotic usage, dosage and course length in children between 0 and 4 years.
Source	Pharmacoepidemiology and Drug Safety (PDS). Conference: 25th International Conference on Pharmacoepidemiology and Therapeutic Risk Management Providence, RI United States. Conference Start: 20090816 Conference End: 20090819. Conference Publication: (var.pagings). 18 (S1) (pp S213-S214), 2009. Date of Publication: August 2009.
Publisher	John Wiley and Sons Ltd
Subject Headings	*child<br>*risk management<br>*pharmacoepidemiology<br>prescription<br>pharmacy<br>data base<br>groups by age<br>recommended drug dose<br>cross-sectional study<br>population<br>*antibiotic agent<br>penicillin derivative<br>amoxicillin<br>clarithromycin<br>acid
Abstract	Background: Antibiotic drugs are most frequently used by 0-4-year-old children. Not all drugs are tested and licensed for this age group and also dosing could be complicated. Objectives: We performed a cross-sectional study using a pharmacy prescription database to investigate the use, dose and course length of antibiotic drugs in 0-4 year olds. Methods: We used IADB.nl, a database with pharmacy drug dispensing data covering a population of 500,000. We investigated all prescriptions of systemic antibiotics prescribed in the years 2002 till 2006 for children of 0-4 years old. Prescriptions for children under the age of 3 months were excluded. Results: Children 9-12 months of age received more antibiotics than children in other age groups. In the 3-6-monthsolds amoxicillin was prescribed in 75.25% of the cases and in the 6-12 month olds in 73.37%. This percentage was in the 4 year olds 50.41%. The contribution of other broadspectrum antibiotics increased with age (clarithromycin and amoxicillin/clavulanic acid). Small-spectrum penicillins were prescribed less often than the broad-spectrum antibiotics. From the prescriptions of the five most used drugs 97.6% were within the recommended dose range. Most course lengths corresponded with the guidelines. Of the prescriptions 3.9% were unlicensed or off-label, most of them for children under 1 year of age. Conclusions: Most antibiotics are used between 9 and 12 months of age. The doses and course lengths are mostly correct. However, the choice of antibiotics was not according to the guidelines. Young children received unlicensed and off-label prescribed antibiotics.
ISSN	1053-8569
DOI	http://dx.doi.org/10.1002/pds.18...
Language	English
Summary Language	English
Publication Type	Journal: Conference Abstract
Entry Week	200900
Date Delivered	20100220
Year of Publication	2009
Copyright	Copyright 2010 Elsevier B.V., All rights reserved.

+ My Projects

2 GO ≫ ◂ Previous | Search Results

Keep Selected

English | Français | Deutsch | 日本語 | 繁體中文 | Español | 簡体中文 | 한국어

Copyright (c) 2000-2012 Ovid Technologies, Inc.

Terms of Use | Support & Training | About Us | Contact Us

Version: OvidSP_UI03.06.00.122, SourceID 56772

Wolters Kluwer Health | OvidSP

My Account | Support & Training | Help | Logoff

Search | Journals | Books | My Workspace

Keep Selected

1 GO ≫ Search Results | Next ▶

1.

Accession Number 2009279792

Authors De Jong J. Van Den Berg P.B. Visser S.T. De Vries T.W. De Jong-Van Den Berg L.T.

Institution (De Jong) Department of Pharmacoepidemiology and Pharmacoeconomics, University of Groningen, Anton Deusinglaan 1, 9713AV Groningen, Netherlands
(De Jong, Van Den Berg, Visser, De Jong-Van Den Berg) Department of Pharmacoepidemiology and Pharmacoeconomics, University of Groningen, Groningen, Netherlands
(De Vries) Medical Centre Leeuwarden, Leeuwarden, Netherlands

Correspondence Address J. De Jong, Department of Pharmacoepidemiology and Pharmacoeconomics, University of Groningen, Anton Deusinglaan 1, 9713AV Groningen, Netherlands. E-mail: josta.de.jong@rug.nl

- Abstract Reference
- Find Similar
- Find Citing Articles
- ubmed findit

Country of Publication United Kingdom

Title Antibiotic usage, dosage and course length in children between 0 and 4 years.

Source Acta Paediatrica, International Journal of Paediatrics. 98 (7) (pp 1142-1148), 2009. Date of Publication: July 2009.

Publisher Blackwell Publishing Ltd (9600 Garsington Road, Oxford OX4 2XG, United Kingdom)

Keyword Antibiotics, Child, Drug dosage calculations, Pharmacoepidemiology, Practice guideline

Subject Headings
*antibiotic therapy
article
child
child health care
clinical practice
*dose calculation
female
general practitioner
human
infant
*infection / dt [Drug Therapy]
major clinical study
male
medical specialist
Netherlands
off label drug use
pharmacoepidemiology
pharmacy
practice guideline
preschool child
prescription
priority journal
respiratory tract infection / dt [Drug Therapy]
*amoxicillin / do [Drug Dose]
*amoxicillin / dt [Drug Therapy]
*amoxicillin plus clavulanic acid / do [Drug Dose]
*amoxicillin plus clavulanic acid / dt [Drug Therapy]
*antibiotic agent / do [Drug Dose]
*antibiotic agent / dt [Drug Therapy]
*azithromycin / do [Drug Dose]
*azithromycin / dt [Drug Therapy]
cephalosporin / do [Drug Dose]
cephalosporin / dt [Drug Therapy]
ciprofloxacin / dt [Drug Therapy]
*clarithromycin / do [Drug Dose]
*clarithromycin / dt [Drug Therapy]
*cotrimoxazole / do [Drug Dose]
*cotrimoxazole / dt [Drug Therapy]
erythromycin / do [Drug Dose]
erythromycin / dt [Drug Therapy]
nitrofurantoin / do [Drug Dose]
nitrofurantoin / dt [Drug Therapy]
penicillin derivative / dt [Drug Therapy]
tetracycline / dt [Drug Therapy]
trimethoprim / do [Drug Dose]
trimethoprim / dt [Drug Therapy]

Abstract Aim: Antibiotic drugs are most frequently used by 0- to 4-year-old children. We performed a cross-sectional study in the Netherlands using a pharmacy prescription database to investigate the use, dose and course length of antibiotic drugs in 0- to 4-year-olds. Methods: We used a database with pharmacy drug-dispensing data. We investigated all prescriptions of systemic antibiotics prescribed in the years 2002-2006 for children of 0-4 years of age. Prescriptions for children under the age of 3 months were excluded. Results: Children of 9-12 months of age received more antibiotics than children in other age groups. In the 3- to 6-month-olds, amoxicillin was prescribed in 75.2% of the cases. This percentage was 50.4% in the 4-year-olds. The contribution of other broad-spectrum antibiotics increased with age (clarithromycin and amoxicillin/clavulanic acid). Small-spectrum penicillins were prescribed less often than the broad-spectrum antibiotics. From the prescriptions of the five most used drugs, 97.6% were within the recommended dose range. Most course lengths corresponded with the guidelines. Of the prescriptions, 3.9% were unlicensed or off-label. Conclusion: Within the group of 0- to 4-year-old children, most antibiotics were used by 9- to 12-month-olds. The doses and course lengths were mostly correct, but the choice of antibiotics was not according to the guidelines. Young children received unlicensed and off-label prescribed antibiotics. 2009 Foundation Acta Paediatrica.

Number of References 21

Embase Section Headings
Pediatrics and Pediatric Surgery [7]
Public Health, Social Medicine and Epidemiology [17]
Drug Literature Index [37]

CAS Registry Numbers 26787-78-0 (amoxicillin); 34642-77-8 (amoxicillin); 61336-70-7 (amoxicillin); 74469-00-4 (amoxicillin plus clavulanic acid); 79198-29-1 (amoxicillin plus clavulanic acid); 83905-01-5 (azithromycin); 11111-12-9 (cephalosporin); 85721-33-1 (ciprofloxacin); 81103-11-9 (clarithromycin); 8064-90-2 (cotrimoxazole); 114-07-8 (erythromycin); 70536-18-4 (erythromycin); 54-87-5 (nitrofurantoin); 67-20-9 (nitrofurantoin); 23843-90-5 (tetracycline); 60-54-8 (tetracycline); 64-75-5 (tetracycline); 8021-86-1 (tetracycline); 738-70-5 (trimethoprim)

ISSN 0803-5253

Electronic ISSN 1651-2227

DOI http://dx.doi.org/10.1111/j.1651...

CODEN APAEE

Language English

Summary Language English

Publication Type Journal: Article

Entry Week 200900

Date Delivered 20090706

Year of Publication 2009

Copyright Copyright 2011 Elsevier B.V., All rights reserved.

+ My Projects

1 GO ≫ Search Results | Next ▶
